# Decoding Early Childhood Caries: A Comprehensive Review Navigating the Impact of Evolving Dietary Trends in Preschoolers

**DOI:** 10.7759/cureus.58170

**Published:** 2024-04-13

**Authors:** Kanika S Dhull, Brahmananda Dutta, Sushmita Pattanaik, Aditi Gupta, Indira MD, Bhushan Wandile

**Affiliations:** 1 Pedodontics and Preventive Dentistry, Kalinga Institute of Dental Sciences, Kalinga Institute of Industrial Technology, Bhubaneswar, IND; 2 Dentistry, Government Medical College and Hospital, Sundargarh, IND; 3 Pediatric Dentistry, Kalinga Institute of Dental Sciences, Kalinga Institute of Industrial Technology, Bhubaneswar, IND; 4 Pedodontics and Preventive Dentistry, JSS Dental College and Hospital, JSS Academy of Higher Education and Research, Mysuru, IND; 5 Medicine, Jawaharlal Nehru Medical College, Datta Meghe Institute of Higher Education & Research, Wardha, IND

**Keywords:** public health, preventive strategies, oral health, dietary trends, preschoolers, early childhood caries

## Abstract

This comprehensive review delves into the intricate relationship between evolving dietary trends in preschoolers and the prevalence of early childhood caries (ECC). The investigation meticulously analyzes ECC epidemiology, etiology, and preventive strategies. The review unveils the multifaceted nature of ECC, highlighting microbial, dietary, and environmental factors contributing to its development. Significantly, the study explores the global prevalence of ECC and its substantial implications for the overall health, nutrition, and development of preschool-aged children. The implications for public health and policy are deliberated, advocating for targeted interventions and collaborative efforts among healthcare professionals, policymakers, educators, and parents. The conclusion presents a compelling call to action, urging collective engagement to mitigate the impact of ECC and prioritize the well-being of preschoolers. This review offers valuable insights for healthcare professionals, policymakers, educators, and parents to inform evidence-based strategies for addressing ECC and promoting early childhood oral health.

## Introduction and background

Early childhood caries (ECC), also known as baby bottle tooth decay or nursing caries, manifests through decayed, missing, or filled tooth surfaces in any primary tooth of a child under six. ECC has emerged as a prevalent and concerning issue on a global scale, ranking among the most common chronic childhood diseases. Its ramifications transcend oral health, impacting a child’s overall well-being, nutrition, and developmental trajectory. Acknowledging the seriousness of ECC is paramount for devising effective strategies to combat this health challenge [1].

Defined as dental issues such as decayed, missing, or filled tooth surfaces in the primary teeth of children aged six and below, ECC demands attention due to its widespread prevalence and potential long-term repercussions [2]. A systematic review of studies conducted in India reveals an alarming prevalence of ECC, ranging from 49.6% to 46.9%. This statistic implies that nearly one in every two children in India is affected by ECC [3]. Notably, Andhra Pradesh exhibited the highest prevalence of ECC at 63%, while Sikkim reported the lowest prevalence at 41.92% [4]. Furthermore, the prevalence of dental caries in the Indian population aged between three and 75 years was 54.16% [5]. However, comprehensive data on the global prevalence of ECC remains unavailable.

The primary objective of this exhaustive review is to delve into the intricate relationship between evolving dietary trends in preschoolers and the prevalence of ECC. By scrutinizing the current knowledge in this domain, it endeavors to comprehensively understand the myriad factors contributing to ECC and pinpoint effective preventive measures. Encompassing a wide-ranging exploration, the review delves into the epidemiology, etiology, and preventive strategies associated with ECC in preschool-aged children. Key objectives include scrutinizing global prevalence and demographic factors influencing ECC, investigating microbial, dietary, and environmental contributors, analyzing evolving dietary trends and their impact on oral health, evaluating the correlation between dietary patterns and changes in oral microbiota, exploring existing preventive measures, and delineating challenges while proposing future directions for research and public health initiatives.

## Review

Epidemiology of ECC

Global Prevalence

Recent studies have underscored the global public health challenge posed by ECC, affecting nearly half of preschool children worldwide [6]. The prevalence of ECC exhibits considerable variation across different countries and populations, with certain regions reporting alarmingly high rates of up to 74.3% among three- to five-year-olds [7]. Notably, ECC disproportionately affects socially disadvantaged populations, with prevalence rates soaring to as high as 85% in some disadvantaged groups [8]. Multiple risk factors contribute to the prevalence of ECC, including feeding and dietary practices, oral hygiene habits, socioeconomic status, and parental attitudes [8]. These findings emphasize the imperative of implementing effective prevention strategies, particularly in areas with high prevalence rates and among vulnerable populations. By addressing these risk factors and tailoring interventions to the specific needs of affected communities, strides can be made toward reducing the burden of ECC and promoting oral health equity.

Demographic Factors Influencing ECC

Several demographic factors have emerged as significant influencers of the prevalence of ECC. Extensive research indicates that sociodemographic variables such as parental education, household income, and maternal psychosocial factors directly impact ECC [9-11]. Furthermore, a study conducted in India revealed that unique risk factors for ECC encompassed the family’s socioeconomic and educational status, the mother’s or caregiver’s oral hygiene practices, and demographic characteristics [12]. Moreover, findings from a separate study indicated that individuals of mixed race and white ethnicity exhibited increased and decreased risks for ECC compared to individuals of black ethnicity, respectively [11]. These insights underscore the critical importance of considering a diverse array of demographic factors in comprehending and addressing the prevalence of ECC across various populations. By acknowledging and addressing these demographic nuances, tailored interventions can be developed to mitigate the burden of ECC within specific communities effectively.

Socioeconomic Impact

Socioeconomic factors have a profound influence on the occurrence of ECC. Children hailing from low-income households and those lacking access to a consistent medical home face heightened susceptibility to dental caries [13]. Notably, ECC disproportionately affects socially disadvantaged populations, with prevalence rates soaring to as high as 85% within certain marginalized groups [14]. Extensive research underscores parental socioeconomic status, educational attainment, household income, and employment status as predisposing factors for ECC [15,16].

Furthermore, a comprehensive study examining the nexus between ECC and poverty in low- and middle-income nations corroborates poverty as a substantial risk factor for ECC [15]. These findings underscore the imperative for deploying effective prevention strategies adept at addressing the socioeconomic determinants associated with ECC. By targeting interventions that specifically address these socioeconomic barriers, strides can be made toward mitigating the burden of ECC, particularly within vulnerable and marginalized populations.

Etiology of ECC

Microbial Factors

Role of *Streptococcus mutans*: *S. mutans *is pivotal in the onset of dental caries, particularly ECC. Distinguished for its robust acidogenic and aciduric properties, *S. mutans* significantly contributes to enamel demineralization [17]. While *S. mutans* is a principal pathogenic agent in dental caries, its presence may exhibit variability, even in children afflicted with severe ECC (SECC). This suggests the potential involvement of other closely associated microbial species in caries progression [17]. Detection of *S. mutans* on tooth surfaces is a robust indicator of cavity development, especially in young children [18]. Furthermore, *S. mutans *has been observed to engage in symbiotic interactions with other microorganisms, such as *Candida albicans*, influencing their cariogenic potential and augmenting the ECC process [19]. Hence, although *S. mutans* remains a pivotal factor in caries initiation, its abundance may not singularly predict caries development, with other microbial entities likely contributing significantly to the decay process [17,19].

Other contributing bacteria: While the etiology of ECC is multifaceted, microbial factors emerge as significant determinants. While *S. mutans* stands out as a primary pathogenic bacterium in dental caries, other bacterial species also assume critical roles in the progression of caries. These encompass various Streptococcus species, including *Streptococcus sanguinis*, low-pH non-*S. mutans* streptococci, and *Atopobium* spp., alongside *Veillonella* spp., *Actinomyces* spp., *Bifidobacterium* spp., and *Lactobacillus fermentum* [17,20,21]. Notably, *Lactobacilli*, in particular, are closely associated with lesion advancement [21]. The dysbiotic state of oral microflora, predominantly instigated by a sugar-rich dietary regimen, is the primary impetus behind ECC [22]. Consequently, while *S. mutans* remains a significant factor in caries evolution, its prevalence alone may not be a solitary predictor, with various other microbial species likely exerting vital roles in tooth decay [17,20,21].

Dietary Factors

Sugar consumption trends: Recent studies highlight sugar consumption as a pivotal factor in developing dental caries among children. The habitual intake of foods and beverages rich in free sugars emerges as a primary catalyst for the onset of dental caries, elevating the risk of ECC [23]. A study on Chinese children aged two to five years revealed associations between ECC and SECC with dietary imbalances, high grain consumption, and limited food variety [24]. Furthermore, household sugar purchases at three years correlate with family sugar consumption, subsequently impacting the incidence of permanent dentition caries [25]. Thus, advocating for reduced free sugar intake and a balanced, diverse diet emerge as fundamental strategies for ECC prevention [23].

Impact of modern dietary habits on ECC: The influence of contemporary dietary patterns on ECC is profound. Infant dietary practices, particularly the consumption of sugary beverages, have been linked to the occurrence of ECC among preschoolers [26]. Notably, a study involving Chinese children aged two to five years revealed that heightened food diversity correlated with reduced caries prevalence [27]. Additionally, frequent consumption of simple carbohydrates, primarily dietary sugars, significantly escalates the risk of dental caries [28]. Moreover, feeding and dietary habits, such as continual sipping or grazing on sugary foods and beverages, have been identified as critical contributors to ECC [12,29]. These findings underscore the importance of promoting healthy, varied dietary habits to safeguard against ECC.

Environmental Factors

Fluoride exposure: Excessive fluoride exposure poses various health risks. Some of the dangers associated with overexposure to fluoride include dental fluorosis, skeletal fluorosis, cardiac insufficiency, reproductive issues, thyroid dysfunction, joint and bone conditions, and neurological problems [30]. Dental fluorosis, characterized by enamel discoloration, stems from elevated fluoride concentrations during childhood. Conversely, skeletal fluorosis manifests as a bone disease, causing discomfort and bone damage. Furthermore, heightened fluoride exposure has been associated with potential health hazards such as decreased fertility, early puberty onset in girls, and neurological disorders, including a potential link to attention deficit hyperactivity disorder [30]. Effective control of fluoride exposure is imperative to mitigate these adverse health effects.

**Figure 1:**
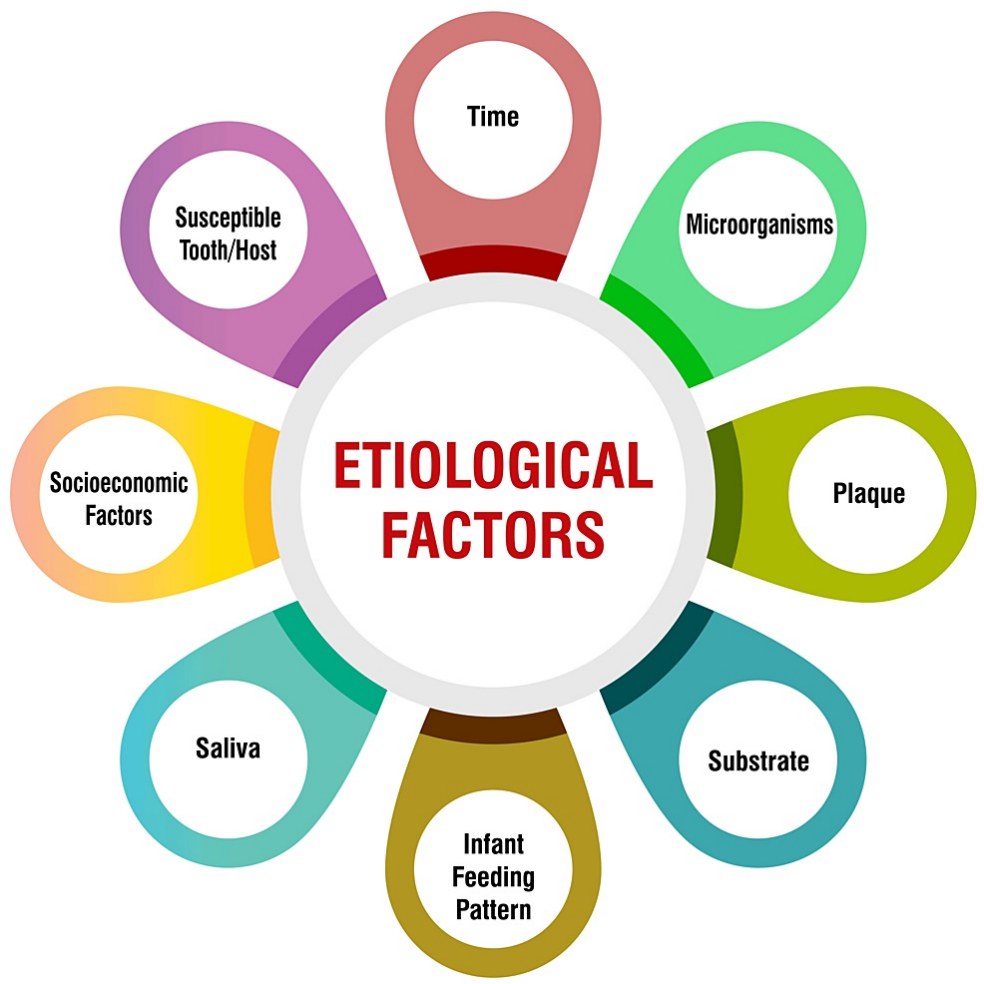
Etiology of ECC ECC, early childhood caries Image credit: Kanika S. Dhull

Socioeconomic and cultural influences: Socioeconomic and cultural determinants significantly impact the prevalence of ECC. Research indicates that children from lower socioeconomic backgrounds are at higher risk of ECC development [9,16,31]. Environmental factors such as culture, lifestyle, and dietary patterns also greatly influence caries susceptibility or resilience [31]. ECC risk factors include inadequate nutrition, suboptimal oral hygiene practices, limited dental care access, and maternal education levels [16]. Additionally, a study investigating nutritional factors associated with ECC underscored the heightened risk posed by increased free sugar consumption [24]. Therefore, addressing socioeconomic and cultural factors and promoting healthy dietary habits and oral hygiene practices is paramount to ECC prevention. Figure 1 shows the etiological factors of dental caries.

Evolving dietary trends in preschoolers

Overview of Contemporary Preschooler Diets

Parents influence their children’s dietary habits significantly, serving as primary role models whose behaviors and eating habits often mirror their own [32]. This parental influence underscores the importance of fostering healthy eating practices within the family. Various social, physical, and intraindividual factors shape children’s eating behaviors, encompassing the family environment, peer influences, and individual preferences [32]. These multifaceted influences necessitate a comprehensive approach to promoting nutritious dietary habits among children. Public health interventions advocate for nutrition education in preschool settings, with a particular emphasis on increasing the consumption of fruits and vegetables [33]. These initiatives aim to cultivate lifelong healthy eating habits by instilling nutritional awareness from an early age. Pediatricians frequently encounter children adhering to various special diets, including vegetarianism, macrobiotics, and exclusion diets for food allergies [34]. Understanding the implications of these dietary patterns is essential for providing tailored medical guidance and support.

The availability of healthy food options, such as fruits, vegetables, and whole grains, alongside the prevalence of fast food and sugary beverages, significantly influences children's dietary choices [35]. Efforts to improve food accessibility and promote healthier alternatives are critical for shaping positive dietary patterns. Emotional eating, characterized by the use of food to assuage emotions or seek comfort, has been linked to increased BMI in young children [35]. Addressing emotional eating behaviors is integral to fostering a healthy relationship with food from an early age. Feeding practices within the family dynamic, including pressure to eat, restrictive feeding behaviors, and monitoring practices, exert notable impacts on children's eating behaviors and food preferences [35]. Recognizing and addressing these practices can facilitate cultivating healthy dietary habits in preschoolers, offering enduring benefits for their health and well-being.

Influence of Processed Foods and Sugary Snacks

Childhood obesity and overweight are often linked to the consumption of sugar-sweetened beverages (SSBs) and ultra-processed foods (UPFs) [36]. The excessive intake of UPFs is known to induce metabolic changes in children and adolescents, further exacerbating the risk of developing overweight or obesity [37]. Processed foods, lacking essential vitamins, minerals, and fiber, frequently contribute to nutritional deficiencies in children [38]. This deficiency arises from the inherent nature of processed foods, which strip away essential nutrients during manufacturing processes.

Moreover, the high sugar and unhealthy fat content prevalent in processed foods can result in excessive calorie intake, leading to weight gain and obesity among children [38]. The disproportionate intake of these calorie-dense foods can disrupt energy balance, consequently fostering unhealthy weight gain. Furthermore, certain artificial additives commonly present in processed foods have been implicated in causing hyperactivity and behavioral disturbances while also potentially impacting neurological function [38]. These additives may exert adverse effects on some individuals, prompting concerns regarding their widespread usage in food products. The appealing taste profile of processed foods, often enhanced by elevated salt, sugar, and fat levels, can lead to addictive consumption patterns [38]. This addictive nature perpetuates overconsumption, thereby exacerbating the risk of obesity and associated health complications.

Additionally, the frequent consumption of sugary snacks and beverages contributes to dental decay and other oral health issues among children [39]. To counteract the adverse effects of processed foods and sugary snacks on preschoolers, it is imperative to foster healthier eating habits and provide alternative, nutritious options [38]. This entails incorporating fresh fruits and vegetables, whole grains, lean proteins, and healthy fats into their diets, ensuring a balanced nutritional intake. Furthermore, parents and caregivers play a pivotal role in modeling healthy eating behaviors and restricting the availability of processed foods within the home environment [36]. By promoting these strategies, efforts can be made to mitigate the negative impact of processed foods on the health and well-being of preschool-aged children.

Beverages and Their Impact on Oral Health

The consumption of sugary drinks can significantly impact oral health. When ingested, the sugars in these beverages fuel bacteria in the mouth, triggering the production of acid that can harm the teeth by causing cavities or erosion. To minimize tooth exposure to the acid produced by bacteria, consuming sweetened beverages in one sitting is advisable rather than sipping them over an extended period. Moreover, if juice is provided to children, it is recommended to have them drink it only with meals and to offer water in a sippy cup for consumption throughout the day. Fluoridated tap water and milk are heralded as superior alternatives for dental health, as they aid in protecting teeth against cavities and maintaining their strength [40]. Research has underscored the association between consuming SSBs and an elevated risk of dental caries and erosion. These findings underscore the detrimental effects of SSBs on oral health outcomes, mainly dental caries and erosion [41]. In contrast, as per the American Dental Association, water is lauded as the optimal beverage for oral health and overall wellness. Additionally, milk is touted as a beneficial option for teeth, as it can safeguard tooth enamel, provide essential vitamins and calcium, and mitigate tooth decay [42].

Cultural and Regional Variations in Dietary Patterns

Cultural and regional variations in dietary patterns are influenced by various factors, such as religious beliefs, food availability, affordability, accessibility, and cultural background. Studies have shown that dietary patterns vary across different regions and cultures, which can impact health outcomes and nutritional status [43-45]. For instance, a study on young Polish females found that the region’s affluence is strongly reflected in dietary behaviors, with higher adherence to traditional Polish dietary patterns in less affluent regions [45]. Similarly, a study on Swiss participants found that statistically significant differences were observed across language regions, with participants in the French- and Italian-speaking regions scoring higher than those in the German-speaking region [46]. These findings highlight the importance of understanding cultural and regional variations in dietary patterns to promote healthy eating habits and prevent diet-related diseases.

Impact of evolving dietary trends on the oral microbiota

Changes in Microbial Composition

Research has delved into the ramifications of evolving dietary trends on the oral microbiota, shedding light on their profound impact. The rapid escalation in carbohydrate consumption, mainly sucrose, has disrupted the evolved equilibrium between the oral microbiota and dental health, rendering dental caries the most prevalent chronic ailment globally [47]. From the advent of agriculture to the Industrial Revolution, dietary shifts have substantially and swiftly escalated carbohydrate intake, unsettling the homeostasis of the oral microbiome and dental well-being [47]. Moreover, studies underscore that diet serves as a vital nutritional source for the oral microbiota while concurrently exerting selective pressure, favoring the survival and propagation of specific organisms. This selective pressure can precipitate pathological alterations in the oral microbiota [48]. Furthermore, research has elucidated that dietary interventions can influence the oral microbiome at the genetic-strain level, impacting the host’s immune response and metabolic profile [49]. Consequently, it is evident that evolving dietary trends profoundly influence the composition and equilibrium of the oral microbiota, with far-reaching implications for both oral and systemic health.

Relationship Between Diet and Bacterial Virulence

The interplay between diet and bacterial virulence is intricate and can yield diverse and sometimes contradictory outcomes. Modifying the host’s diet has the potential to either suppress or exacerbate disease severity and the proliferation of pathogens [50]. For instance, epidemiological investigations have highlighted a correlation between diet and the risk of gastric cancer, particularly concerning* Helicobacter pylori* infection [51]. Moreover, pathogens operate within dynamic nutritional microenvironments within the host, and the host’s diet can influence microbial virulence, a phenomenon termed “nutritional virulence” [52]. Consequently, the impact of diet on bacterial virulence encompasses many factors, including the host’s immune response, pathogen adaptability, and the diversity and functional capacity of the microbial community [53].

The Role of Diet in Biofilm Formation

Dietary sugars have been identified as critical regulators of bacterial-fungal interactions in saliva, impacting interkingdom biofilm formation on tooth surfaces. Research indicates that sucrose and starch facilitate the coexistence of bacteria and fungi, fostering heightened biofilm accumulation and acid production, potentially contributing to ECC development [54]. Moreover, forming biofilms in the food industry constitutes a multifaceted process wherein the quantity and composition of nutrients, including dietary components, influence biofilm development [55]. Furthermore, investigations into the impact of diet on oxidative stress and inflammation induced by bacterial biofilms in the oral cavity underscore the role of specific dietary patterns in shaping biofilm induction and the proliferation of both pathogenic and beneficial bacteria [56]. These findings underscore the significant role of diet in modulating biofilm formation and its potential ramifications for both oral and systemic health.

Preventive measures and interventions

Importance of Early Oral Health Education

Early oral health education is pivotal in fostering positive oral health habits and averting dental caries in children. The research underscores the necessity of commencing health education at an early age to monitor growth and stave off potential pathologies [57]. It is worth noting that subpar oral health can translate to absenteeism from school and diminished academic performance among children [58]. Regular preventive dental checkups with oral health professionals facilitate the dissemination of age-appropriate anticipatory guidance to parents and caregivers [59]. Many countries have implemented oral health education programs within school settings, recognizing early childhood as a critical phase for optimal oral health [57]. The overarching aim of oral health education is to enhance knowledge, which fosters the adoption of favorable oral health behaviors conducive to improved oral well-being [60]. Furthermore, various preventive measures and interventions have been identified for ECC prevention. These include regulating fermentable carbohydrate intake, avoiding bottle or sippy cup usage before bedtime, ensuring adequate fluoride exposure, scheduling regular preventive dental appointments, disseminating oral health education, implementing screening strategies, and fostering interprofessional collaboration [59]. By employing these multifaceted approaches, efforts can be concerted toward mitigating the burden of ECC and promoting enduring oral health among children.

Promoting Healthy Dietary Habits

Promoting healthy dietary habits encompasses a range of strategies and approaches advocated by various reputable sources. The CDC advocates the “Reflect, Replace, Reinforce” method, which entails introspection on eating habits, the substitution of unhealthy choices with healthier alternatives, and the reinforcement of new habits [61]. Additionally, the CDC recommends meal planning, eating well-balanced meals, and exercising patience when transitioning to new eating patterns [61]. Furthermore, the WHO stresses the importance of fostering healthy nutrition throughout all stages of life and establishing a conducive food environment through collaborative efforts involving multiple sectors and stakeholders, including government bodies, the public, and the private sector [62]. The WHO also recommends decisive actions by policymakers, such as harmonizing trade, food systems, and agricultural policies, to encourage healthy dietary practices [61]. Moreover, resources like the Early Childhood Learning and Knowledge Center (ECLKC) and KidsHealth offer practical guidance for families, including serving a diverse array of nutritious foods and snacks, curbing the intake of sugary beverages, and involving children in the food selection process [63,64]. These resources underscore the significance of adopting a holistic approach to promoting healthy dietary habits, encompassing individual reflection, environmental support, and policy-level interventions.

Role of Fluoride in Prevention

Fluoride is a cornerstone in preventing dental caries among children [65]. Its mechanisms include bolstering dental mineralization and bone density, exhibiting bactericidal effects on cariogenic bacteria, and retarding demineralization while fostering enamel remineralization within dental plaque [66]. Fluoride varnish emerges as a well-established tool for preventing ECC, renowned for its ease of application and favorable tolerance by children [67,68]. Other modalities for fluoride administration in caries prevention encompass fluoride toothpaste, prescription fluoride supplements, and fluoride mouth rinses [65,69]. Nonetheless, while fluoride’s efficacy is widely acknowledged, evidence regarding the effects of various fluoride concentrations remains somewhat limited, necessitating consideration of the risk of dental fluorosis across different fluoride concentrations [66].

Dental Sealants and Their Efficacy

Dental sealants, thin coatings applied to the chewing surfaces of the back teeth (molars), protect against cavities [70]. Proven effective, they are instrumental in preventing and halting pit-and-fissure occlusal caries lesions in both primary and permanent molars among children [71]. Additionally, sealants can impede the progression of noncavitated occlusal caries lesions [72]. Research indicates resin sealants offer a preventive fraction of up to 61% over five years [72]. These sealants can be categorized into three types: glass ionomer, resin-modified glass ionomer, and resin-based sealants, with the latter preferred for their superior retention and effective caries prevention [72]. Studies demonstrate that dental sealants can reduce the incidence of dental caries by up to ninefold [73], making them a cost-effective intervention, particularly for children at high risk of developing cavities [73]. Sealants can prevent 80% of cavities in the back teeth over two years, a significant statistic given that nearly nine out of 10 cavities occur in these teeth [70]. Despite their efficacy, the utilization of sealants remains suboptimal, with less than half of children and adolescents benefiting from this preventive measure [70]. Closing this gap in sealant utilization could significantly enhance oral health outcomes among young populations.

Challenges in addressing ECC

Lack of Awareness and Education

Several factors contribute to the prevalence of ECC, necessitating multifaceted approaches for prevention and intervention. Firstly, a notable need for more awareness among parents and caregivers regarding the significance of early oral health and the potential repercussions of ECC persists [2]. This deficiency in awareness often translates into inadequate oral hygiene practices and inappropriate feeding habits, further exacerbating the risk of ECC development [2]. Secondly, there is a pressing need for more comprehensive education on oral health and ECC prevention targeted at parents, caregivers, and healthcare professionals [1]. This educational endeavor should emphasize the importance of oral hygiene, dietary habits, and the detrimental effects of improper feeding practices [1]. Moreover, socioeconomic and educational factors significantly influence ECC prevalence, with socially disadvantaged populations and children from low-income families being disproportionately affected [2]. Addressing these socioeconomic and educational determinants emerges as pivotal in mitigating the prevalence of ECC. Additionally, enhancing integration between medical and dental healthcare systems is imperative for delivering preventive services and fostering interdisciplinary approaches to oral health promotion [74]. Cultural factors also play a pivotal role, with parents’ education levels, stress levels, oral health beliefs, attitudes, and cultural backgrounds closely intertwined with ECC and dental caries [75]. Addressing these cultural nuances holds promise for improving oral health outcomes among children. To tackle these challenges comprehensively, concerted efforts should be made to augment awareness and education on oral health and ECC prevention, enhance access to dental care, and promote interdisciplinary approaches to oral health promotion [1,2,74]. This endeavor encompasses developing and implementing evidence-based prevention strategies, integrating oral health education into school and community settings, and enhancing access to dental services for socially disadvantaged populations.

Barriers to Accessing Dental Care

The hurdles encountered in tackling ECC encompass obstacles to accessing dental care, which can significantly impact children's health and overall well-being. Untreated cavities in children can lead to pain, infections, and difficulties with essential activities such as eating, speaking, playing, and learning [76]. Hence, access to dental care emerges as a critical component in preventing and managing ECC. The research underscores the significance of harnessing technology to reach vulnerable families and assist them in cultivating positive oral health behaviors aimed at averting tooth decay in young children [76]. Furthermore, factors including excessive sugar consumption, poor oral hygiene practices, inadequate fluoride exposure, and enamel defects play substantial roles in ECC development, underscoring the necessity for preventive measures and enhanced access to dental care [2]. Effectively addressing disparities in early childhood dental caries mandates integration between medical and dental healthcare systems to provide preventive services within primary healthcare settings [74]. Moreover, parental challenges in implementing oral health practices are influenced by various factors such as education, stress levels, health beliefs, attitudes, and cultural considerations [75]. These findings underscore the paramount importance of surmounting barriers to dental care access and implementing efficacious strategies for preventing and managing ECC.

Cultural and Socioeconomic Challenges

**Figure 2:**
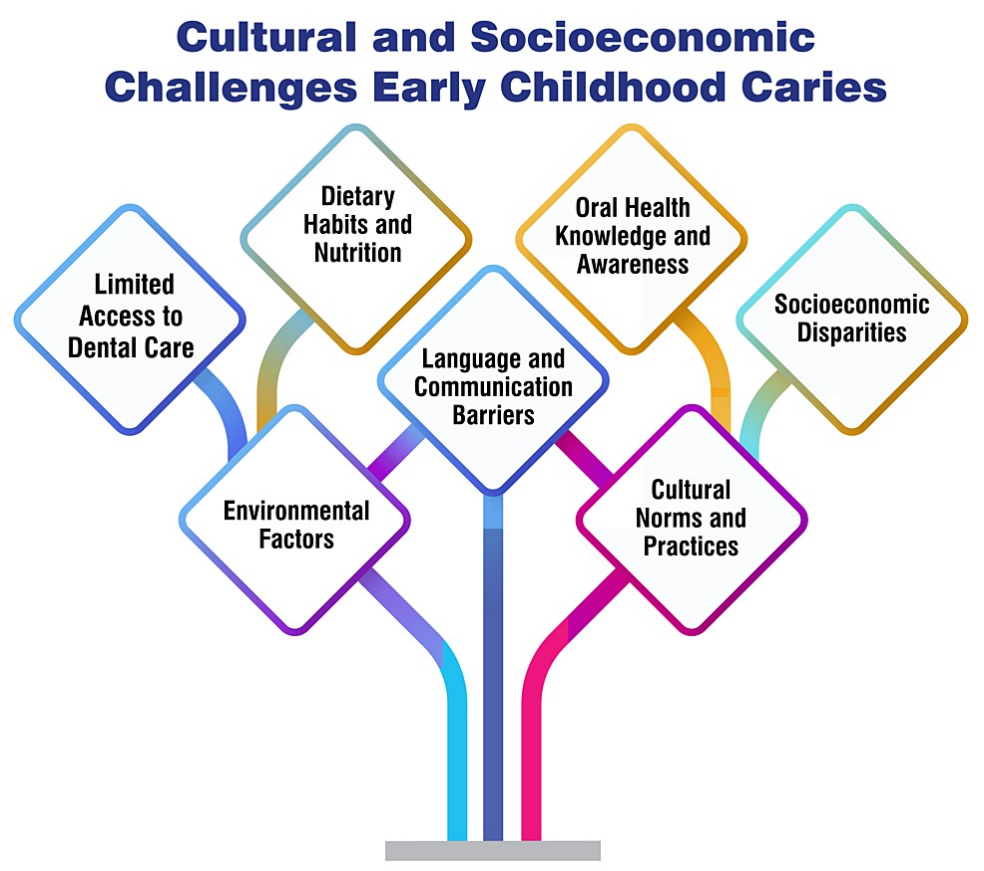
Cultural and socioeconomic challenges in ECC ECC, early childhood caries Image credit: Kanika S. Dhull

Addressing ECC poses challenges influenced by diverse cultural and socioeconomic factors. Recent research has underscored the profound impact of factors such as excessive sugar consumption, low maternal education levels, and varying socioeconomic statuses on the susceptibility to dental caries among children in low- and middle-income countries [77]. Additionally, ECC risk is closely linked to factors like feeding practices, dietary habits, oral hygiene routines, and limited access to dental care, particularly among socially disadvantaged populations [2,12]. Furthermore, it has been highlighted that families significantly influence the dissemination of health-related information regarding oral health. Thus, interventions targeting individual, familial, and communal levels effectively address ECC [1]. Moreover, a comprehensive review of oral health policies across different regions has underscored the necessity for holistic strategies to alleviate the ECC burden while considering the cultural and socioeconomic determinants of the disease [78]. These findings underscore the imperative of tailored interventions for the diverse cultural and socioeconomic contexts inherent in preventing and managing ECC. By addressing these multifaceted factors, efforts to combat ECC can be rendered more effective and inclusive, ultimately promoting improved oral health outcomes among children. Cultural and socioeconomic challenges in ECC are shown in Figure 2.

## Conclusions

This comprehensive review has revealed significant insights into the complex dynamics of ECC. Exploring the intricate relationship between evolving dietary trends in preschoolers and ECC prevalence has shed light on the multifaceted nature of this oral health concern. The findings underscore the global prevalence of ECC and its multifactorial origins, encompassing microbial, dietary, and environmental influences. The implications for public health and policy are substantial, emphasizing the urgent need for targeted interventions and preventive measures at both community and policy levels. The review advocates for a collaborative approach involving healthcare professionals, policymakers, educators, and parents to formulate and implement effective strategies. Furthermore, the call to action extends to healthcare professionals for knowledge dissemination and policy advocacy, educators integrating oral health education into curricula, and parents actively participating in their children’s oral health practices. By fostering such collaborative efforts, we can work toward a future where ECC is minimized and the well-being of preschoolers is prioritized both dentally and holistically. This call to action serves as an invitation to unite in the collective pursuit of a healthier and brighter future for the youngest members of our communities.
